# Shearlets as feature extractor for semantic edge detection: the model-based and data-driven realm

**DOI:** 10.1098/rspa.2019.0841

**Published:** 2020-11-25

**Authors:** Héctor Andrade-Loarca, Gitta Kutyniok, Ozan Öktem

**Affiliations:** 1 Institut für Mathematik, Technische Universität Berlin, 10623 Berlin, Germany; 2 Fakultät Elektrotechnik und Informatik, Technische Universität Berlin, 10587 Berlin, Germany; 3 Department of Physics and Technology, University of Tromsø, Tromsø, Norway; 4 Department of Mathematics, KTH - Royal Institute of Technology, SE-100 44 Stockholm, Sweden

**Keywords:** multiscale geometric analysis, harmonic analysis, deep learning, feature extraction

## Abstract

Semantic edge detection has recently gained a lot of attention as an image-processing task, mainly because of its wide range of real-world applications. This is based on the fact that edges in images contain most of the semantic information. Semantic edge detection involves two tasks, namely pure edge detection and edge classification. Those are in fact fundamentally distinct in terms of the level of abstraction that each task requires. This fact is known as the distracted supervision paradox and limits the possible performance of a supervised model in semantic edge detection. In this work, we will present a novel hybrid method that is based on a combination of the model-based concept of shearlets, which provides probably optimally sparse approximations of a model class of images, and the data-driven method of a suitably designed convolutional neural network. We show that it avoids the distracted supervision paradox and achieves high performance in semantic edge detection. In addition, our approach requires significantly fewer parameters than a pure data-driven approach. Finally, we present several applications such as tomographic reconstruction and show that our approach significantly outperforms former methods, thereby also indicating the value of such hybrid methods for biomedical imaging.

## 1. Introduction

In computer vision, semantic edge detection aims to detect edges and object boundaries in natural images and to classify the points in those objects, which is of significant importance in various applications such as object recognition, semantic segmentation and image reconstruction. Examples of such classification could be to label each point of an edge according to the object this edge belongs to [1] or the orientation of the edge at that particular point [2]. Thus, this problem combines two different classification tasks. The first is classical category-agnostic edge detection, which can be viewed as a pixel-wise binary classification for determining whether a pixel belongs to an edge or not. The second is the classification of those pixels into prescribed classes.

Previous approaches to performing semantic edge detection can be divided into two main groups, namely model-based and data-driven methods; each has its own strengths and shortcomings. The main idea presented in our paper is to overcome these shortcomings by combining elements of model-based and data-driven approaches for semantic edge detection. We remark that this objective follows a common thread of current research, aiming for an optimal combination of model-based or data-driven methodologies.

Our conceptually general approach aims to combine advances from several fields. First, it uses properties of the representation systems of shearlets, foremost to optimally approximate (and detect) singularity structures [3]. Second, it also takes advantage of microlocal analysis for describing how singularities transform when acted upon by a wide range of operators [4]. Finally, it also leverages on the proven track record that convolutional neural networks (CNNs) have had in image classification [5].

### (a) Why is semantic edge detection important?

The concept of edge detection is one of the principal problems in the field of image processing and computer vision. This is because edges in images represent the boundaries of objects and carry most of the information for the associated physical scene [6–9]. It has also been shown in [8] that the human visual cortex performs multiple operations of image processing, the first of which is rough sketching, which involves edge detection in order to reduce the amount of information that needs to be fully acquired given that the visual cortex is sparsely connected.

Based on this importance, pure edge detection is used in a wide range of applications in computer vision and other fields. For example, classical optical flow algorithms that are used for object tracking, which exploit stable-in-time features such as corners—corners being a particular case of edge points—in order to track the objects containing those. This can, for instance, be used in turn for object depth estimation [10] and three-dimensional image reconstruction.

Semantic edge detection has a deeper impact in computer vision than solely edge extraction. Assigning, for instance, class labels to edge points corresponding to the object that the respective edge belongs to is crucial for applications such as object proposal generation [11], occlusion reasoning [12], object detection [13,14] and image-based localization [15].

An important special case of semantic edge detection is to classify edge points according to the orientation of the respective edge in that pixel. This is also termed wavefront set extraction, owing to the fact that the microlocal wavefront set precisely consists of—coarsely speaking—singularity points together with their direction. Wavefront set extraction is widely applied, in particular, in inverse problems such as these, where the forward operator is a Fourier integral operator. These operators occur in biomedical imaging problems such as computed tomography and magnetic resonance imaging.

### (b) Model-based semantic edge detection

Classical model-based approaches for edge extraction usually involve two steps: a filtering step to enhance edge-like features and a classification step to identify pixels belonging to edges. The filtering step typically follows simple rules and heuristics, e.g. convolutions with local difference filters correspond to operating on the image with Roberts [16], Sobel [17] and Prewitt [18] operators. In a similar manner, the well-known Canny edge detector [19] corresponds to convolving the image with a Gaussian kernel to further identify those pixels where the gradient has local maxima. There have been attempts in the past to also model the semantic information of detected edges, as in [20]. This work was in fact among the first publications to propose a principled way to combine generic object detectors with bottom-up contours for semantic edge detection.

Focusing on the special case of wavefront set extraction, one class of approaches is based on systems from applied harmonic analysis such as shearlets. An approach of this type is the shearlet-based algorithm in [21], which uses digital shearlets to filter an image in order to highlight the features corresponding to different orientations and scales. One then performs a simple unsupervised classification algorithm based on clustering (e.g. k-means) to classify the corresponding directions. A more recent approach is [22,23], where a general directional system, known as symmetric molecules, is used to filter the directional features of images to then classify them as belonging to the class edge, ridge or blob.

These model-based approaches for semantic edge detection rely on ‘first principles’ from approximation theory and are easy to interpret. On the other hand, the use of rigid heuristics regarding the characterization of singularities makes it difficult to use these methods in real-world applications, where the data represent empirically defined function classes.

### (c) Multiscale directional systems as model-based feature extractors

The shearlet system plays a unique role as a multiscale directional system in imaging applications as a feature extractor, therefore it will also be the key ingredient of the model-based side of our hybrid approach in this paper. In contrast to the isotropic system of wavelets [24], curvelets [25] were the first system to provably provide optimally sparse representation of curvilinear structures. However, the use of the rotation operator to parameterize directionality was one main reason which prevented implementation that was consistent with the continuum theory [26].

With the introduction of shearlet systems in [3] based on shearing as a directionality measure, these limitations could be overcome, leading to a uniform concept for the continuum and digital realm [27]. In addition, compactly supported shearlets could be shown to provide optimally sparse approximations of curvilinear structures as well, thereby allowing high spatial localization [28]. Of particular benefit for semantic edge detection is the ability of continuous shearlet systems to characterize the wavefront set through the asymptotic decay of the associated shearlet coefficients [29], with properties resembling the discrete shearlet systems [30].

### (d) Data-driven semantic edge detection

More recently, machine learning methodologies swept the fields of imaging sciences and computer vision with tremendous success, mainly because physical models for natural images do not exist, thereby making data-driven methods highly effective. These modern approaches have been recently adopted for category-agnostic edge detection. A prominent example was proposed by Liu *et al.* [31] by the introduction of the so-called richer convolutional features, which encapsulate convolutional features coming from layers of deep CNNs into more discriminative representation; this overcomes the issue of deep CNNs failing to capture complex data structures with wide variations of scale, imposing a state of the art. With a similar aim, Xu *et al.* [32] introduced a multi-scale feature extractor for accurate contour detection based on multiscale representations encoded by CNNs.

This trend can also be observed for the application of semantic edge detection, where deep neural networks have led to a new state of the art of semantic edge detection [1,11,33,34]. In fact, these methods exploit similar principles to their model-based counterparts, such as learning filters—now by using convolutional layers—and subsequently classifying the corresponding edge pixels—now by sigmoid or softmax classifiers. Delving deeper into the architecture of a CNN, one can observe that each convolutional layer represents a level of abstraction of the features in the target images: the initial layers will represent ‘simple’ edges, whereas deeper layers will represent more ‘complex’ features.

This consideration shows that in the case of data-driven approaches—but also in general—the two steps involved in semantic edge detection, namely semantic-agnostic edge detection and edge classification (cf. the beginning of §1), are conceptually far from each other. Therefore, no straightforward way exists to jointly learn how to extract and classify the edges. This limitation in semantic edge detection is known as the ‘distracted supervision paradox’ [33].

### (e) The distracted supervision paradox

Semantic-agnostic edge detection involves locating finely detailed edges by capturing discontinuities among image regions, which mainly makes use of low-level features in a CNN. Moreover, edge classification requires high-level semantics to be identified by summarizing different appearance variations in the target classes. This so-called distracted supervision paradox was introduced by [33] and prevents the state-of-the-art end-to-end semantic edge detection method based on deep supervision.

Given the seriousness of this limitation, researchers have tried non-standard ways to avoid the distracted supervision paradox. One idea is to directly use high-level convolution features for semantic classification and low-level convolutional ones for non-semantic edge detection by jointly optimizing over the two corresponding losses. In [1], the authors proposed the CASENet architecture based on a concatenation of convolutional residual networks, where category-wise edge activations in the top layers are shared and fused with the same set of bottom layer features. This architecture introduced a new state of the art in semantic edge detection and can be considered a milestone in the field. However, the authors also showed that such an approach actually decreases the performance compared with directly learning semantic edges without deep supervision. More recently, a new training approach for CASENet was proposed in [34], the simultaneous edge alignment and learning (SEAL) method. The SEAL method simultaneously aligns ground truth edges and learns semantic edge detectors. Inspired by these approaches, [33] proposed an end-to-end architecture using the CASENet backbone with an information-converter layer to transform information coming from low-level features and change it into different representations. This approach is known as *diverse deep supervision* and has shown an improvement in performance with respect to the two previous architectures.

The main drawback of these approaches lies in the complicated layers of the related deep neural network architectures, which are designed as an elaborate way to avoid the distracted supervision paradox. In addition, owing to the large number of network parameters, these methods are slow and difficult to train.

For the special case of wavefront set extraction (see §1a), where the class an edge point belongs to represents the orientation of the edge in that particular point, assuming a finite set of orientations, a hybrid approach was suggested in [2]. This method is based on a careful combination of the shearlet transform and a CNN. It came as a surprise that a significantly smaller architecture was apparently sufficient to obtain state-of-the-art results in semantic edge detection.

### (f) Our contributions

The main goal of this paper is not primarily the introduction of entirely new methods, but a combination of known seemingly disconnected approaches to improve their overall performance and increase the interpretability of the results. In this sense, the main contributions of our work are threefold.

— *Hybrid semantic edge detection.* We introduce the first hybrid method for the general problem class of semantic edge detection. Our approach carefully combines the model-based approach of the shearlet transform with the data-driven approach of deep CNNs, where one key ingredient is to apply the neural networks in a more convenient domain of image representation. In order to evaluate our novel method, we present benchmarks on the human-annotated semantic boundaries dataset (SBD) [35] and a custom dataset made of random ellipses and analytically defined semantic edges.
— *Avoidance of the distracted supervision paradox.* Our hybrid approach avoids the distracted supervision paradox, but at the same time requires significantly fewer parameters than a pure data-driven approach, leading to a highly efficient methodology.
— *Application of semantic edge detection to computed tomography.* We show how the so-called micro-canonical relation, which prescribes the relation of the wavefront set of an image to the wavefront set of its transformation under a forward model, can be used in combination with our hybrid approach to efficiently solve the computed tomography problem. In fact, we strongly expect this conceptual approach to also be useful in other inverse problem settings.

### (g) Overview of the paper

In §2, we introduce the main concepts of microlocal analysis such as wavefront sets, in order to provide an abstraction of edges as singularities of distributions. We also discuss their importance for inverse problems, in particular for two-dimensional (2D) edge detection. This is followed by the presentation of the algorithmic way to compute the wavefront set introduced in [2] by the authors, which also requires recalling key notions related to shearlets and CNNs (see §3). Furthermore, we show that digital wavefront set extraction is a special case of the semantic edge detection problem and discuss in which sense this method avoids the distracted supervision paradox. In §4, we then introduce a hybrid approach for general semantic edge detection based on the approach discussed in §3. The shearlet representation is used for heavy lifting in semantic-agnostic edge detection, thereby improving semantic classification as well as complexity. In addition, we introduce two architectures which implement these ideas based on already known architectures for semantic edge detection, resulting in better performance with fewer parameters. Section 5 is devoted to numerical experiments that show the advantages of using the model-based shearlet representation in semantic edge detection. We also present an application of wavefront set extraction in computed tomography reconstruction. Finally, we contemplate our conceptual approach and numerical results in §6.

## 2. Microlocal analysis

### (a) Basic definitions and properties of distributions

Many notions, like the ‘density of a point mass’, are difficult (or impossible) to represent as functions in classical analysis. Furthermore, one often also seeks to study the propagation of singularities of fundamental solutions to partial differential equations, which in a classical function setting would require differentiating functions that are not necessarily differentiable.

Distribution theory was developed to meet these needs; in the following, we provide a very brief description of this theory. Our emphasis is on the role of distributions in studying the propagation of singularities under various types of operators (differential, pseudo-differential or Fourier integral), a field commonly referred to as microlocal analysis. A more complete detailed exposition of distribution theory can be found in [36,37].

The space of distributions, $D^{\prime }(\Omega )$, is the topological dual (with the weak-* topology) of the Fréchet space $D(\Omega ):=C_{0}^{\infty }(\Omega )$ (test functions). Hence, a distribution $u\in D^{\prime }(\Omega )$ is a continuous linear functional on D(Ω). Another commonly used space of distributions is $E^{\prime }(\Omega )$, which is the topological dual of $E(\Omega ):=C^{\infty }(\Omega )$. Note that any locally integrable function f:Ω→R can be identified with a distribution $u_{f}\in D^{\prime }(\Omega )$ given as

$$u_{f}(\phi ):=\int_{\Omega }f(x)\phi (x)\;\text{d}x\;\text{for any }\phi \in D(\Omega ).$$

There also exist distributions which do not correspond to a function. The simplest example is the Dirac *δ*-distribution at a point *x*_0_ ∈ *Ω*, which is defined as $\delta_{x_{0}}:D(\Omega )\to R$ with $\delta_{x_{0}}(f):=f(x_{0})$.

A central property of distributions is that they can often be arbitrarily differentiated and the result is again a distribution. More precisely, the partial derivative ∂^*α*^ *u* of $u\in D^{\prime }(\Omega )$ is defined as (∂^*α*^ *u*)(*f*) : = ( − 1)^|*α*|^ *u*(∂^*α*^ *f*) for f∈D(Ω) and multi-index *α*. In particular, any locally integrable function has a distributional derivative.

One can also extend the notion of a Fourier transform to distributions. To do this, we start by introducing the Schwartz (tempered) distributions $S^{\prime }(R^{n})$ as the topological dual of the Schwartz space $S(R^{n})$, which consists of smooth functions whose derivatives decrease rapidly. The Fourier transform is an automorphism on this space, so we can define the Fourier transform of a tempered distribution $u\in S^{\prime }(R^{n})$ as $\hat{u}(f):=u(\hat{f})$ for $f\in S(R^{n})$. By duality (using the Plancherel formula) one can show that the above defined Fourier transform operator extends to a weak-* continuous linear map from $S^{\prime }(R^{n})$ to $S^{\prime }(R^{n})$.

### (b) The wavefront set

As already indicated, microlocal analysis is the detailed analysis of how singularities of distributions propagate under various operators. Such an analysis requires one to extend the notion of singular support, which brings us to the wavefront set that describes simultaneously the location and ‘direction’ of the singularities.

The wavefront set is based on the Fourier transform characterization of singularities. More precisely, the starting point is the well-known fact that a compactly supported function is infinitely differentiable if and only if its Fourier transform decays as *O*(|*ξ*|^−*m*^) as |*ξ*| → ∞ for every *m* = 1, 2, …. This characterizes the singular support $\mathrm{sing}\;\mathrm{supp}(f)\subset R^{n}$, which is defined as the complement of the largest open set where *f* is *C*^∞^.

The singular support is however not invariant under a smooth change of coordinates, which makes it difficult to use it for understanding how singularities are transformed. The key observation is to perform a further localization (micro-localization) that identifies the directions at a singular point where the localized Fourier transform does not decay sufficiently fast. An element in the wavefront set is a pair consisting of a point in the singular support and a direction that causes the singularity. This becomes a subset of $R^{n}\times S^{n-1}$ for $f\in D^{\prime }(R^{n})$, where the unitary vectors in *S*^*n*−1^ represent the directions ‘causing’ the singularity. However, in many applications like tomographic imaging, one needs to consider distributions on a manifold *M*. To define the wavefront set for such distributions we represent the aforementioned direction as an element in the cotangent space of *M* at the point in the singular support. The formal definition reads as follows.

**Definition 2.1.**

Let *M* be a smooth manifold and $f\in D^{\prime }(M)$. We say that *f* is *microlocally smooth* at $\left(x_{0},\xi_{0})\in T^{*}(M)\setminus \{0\right\}$ if there exists a neighbourhood *U* ⊂ *V* of *x*_0_ and $\psi \in C_{0}^{\infty }(U)$ with *ψ*(*x*_0_) ≠ 0 and a conic neighbourhood *Γ* of *ξ*_0_ such that for constants *C*_*m*_ we have

2.1 $$\left|(\hat{\psi f})(\xi )\right|\leq C_{m}{\left(1+|\xi |\right)}^{-m}\;\text{for }\xi \in \Gamma \text{ with }m=1,2,\ldots .$$

The **C*^∞^-wavefront set* $\mathrm{WF}(f)\subset T^{*}(M)\setminus \{0\}$ is the set of (*x*_0_, *ξ*_0_), where *f* is not microlocally smooth.

To see an example, let $f\in D^{\prime }(R^{n})$ be a smooth density on the hyper-surface *x*_*n*_ = 0, i.e. *f*(*x*) = *f*(*x*′, *x*_*n*_) = *g*(*x*′)*δ*_0_(*x*_*n*_) for some $g\in C^{\infty }(R^{n-1})$. Then

$$\mathrm{WF}(f)=\{(x^{\prime },0;0,\xi_{n}):x^{\prime }\in \mathrm{supp}(g)\text{ and }\xi_{n}\neq 0\}.$$

Another example is when *f* is the characteristic function of a domain $\Omega \subset R^{n}$ with smooth boundary. Then WF⁡(f) is

WF⁡(f)={(x,ξdx):x∈∂Ω and ξ is normal to ∂Ω at x}.

Furthermore, if *f* is a linear combination of characteristic functions on sets with smooth boundaries, then WF⁡(f) is the union of the co-normal bundles to the individual sets unless cancellation occurs along shared boundaries. Further examples are given in [4, §4].

### (c) Characterization of visible singularities in tomography

Having defined the notion of a wavefront set, we now turn our attention to characterizing how an operator transforms the wavefront set in the context of inverse problems.

Letting *M* be a smooth manifold, we seek to study how an operator $P:D^{\prime }(M)\to D^{\prime }(M)$ transforms the wavefront set. One can show that if *P* is a differential operator, then it does not increase the wavefront set, i.e. it does not add singularities. This is a special case of a more general result that is proven to also be the same when *P* is a pseudo-differential operator [4, theorem 14],

sing supp⁡(P(f))⊂sing supp⁡(f)andWF(P(f))⊂WF⁡(f)

with equality if *P* is in addition elliptic, i.e.

sing supp⁡(P(f))=sing supp⁡(f)andWF(P(f))=WF⁡(f).

Hence, a pseudo-differential operator may spread the support of a function *f*, but it does *not spread* its singular support or *wavefront set*. Consider next the more general case when $P:D^{\prime }(M)\to D^{\prime }(N)$ is a Fourier integral operator [4, definition 7]. The relation between the wavefront sets of *P*(*f*) and that of *f* is then given by the Hörmander–Sato lemma

2.2 $$\mathrm{WF}\left(P(f)\right)\subset C\circ \mathrm{WF}(f)\;\text{for }f\in E^{\prime }(M).$$

In the above, *C* ⊂ T*(*N*) × T*(*M*) is the (microlocal) canonical relation associated with *P*; see [4, eqn (52)] for its formal definition. These results for pseudo-differential and Fourier integral operators are referred to as the *pseudo-local property*.

We are now interested in applying the above theory to inverse problems, such as tomographic imaging. The aim here is to recover a distribution/function *f* from noisy realizations of *g*: = *P*(*f*) (data), where *P* is some known operator (forward operator). *P* is in many applications a pseudo-differential operator, or more generally a Fourier integral operator. The pseudo-local property can then be used to relate the wavefront sets of *g* and *f*. Next, a (regularized) inverse of *P* is typically defined by some related operator *Q*, which is often a Fourier integral operator. Again, one can use the pseudo-local property to relate the wavefront sets of *Q*(*g*) for *g* = *P*(*f*) and *f*. This can guide the design of a *Q* that allows the singularities of *f* to be recovered.

In the following, we illustrate the above in the context of image reconstruction in planar X-ray transmission tomography. We can model data in a simplified setting as samples of the 2D ray transform and image reconstruction amounts to inverting this transform. The ray transform is an integral transform that maps $f\in D^{\prime }(R^{2})$ to a function/distribution *g* on lines in $R^{2}$. Introducing coordinates on the manifold of lines in $R^{2}$ allows us to explicitly define the operator. Any line $\ell \subset R^{2}$ can be described uniquely by (θ,p)∈[0,π]×R through

$$\ell =\left\{x\in R^{2}:x=s\mapsto p\omega (\theta )+s\omega {\left(\theta \right)}^{⊥}\right\},\;\text{where}\;\left\{ \begin{array}{ll} \omega (\theta ):=(\cos\theta ,\sin\theta )\in S^{1}, & \\ \omega {\left(\theta \right)}^{⊥}:=(-\sin\theta ,\cos\theta ). & \end{array}\right.$$

Hence, (θ,p)∈[0,π]×R serves as coordinates for a sub-manifold M of lines in $R^{2}$, i.e. M⊂[0,π]×R. Using these coordinates, we can express the ray transform of $f\in D^{\prime }(R^{2})$ as

$$g=R(f)(\theta ,p):=\int_{-\infty }^{\infty }f\left(p\omega (\theta )+s\omega {\left(\theta \right)}^{⊥}\right)\;ds\;\text{for }(\theta ,p)\in M.$$

The aim in tomographic imaging is to reconstruct *f* from *g* = *R*(*f*), preferably by using a stable recovery scheme. A closely related task is to recover the wavefront set of *f* from the wavefront set of data $g\in D^{\prime }(M)$, which is a subset of $T^{*}(M)$. The 2D ray transform *R* is a Fourier integral operator (see [4, corollary 1]), so there is a micro-canonical relation as in (2.2). The following theorem characterizes these singularities of $f\in D^{\prime }(R^{2})$, which can be recovered from ray transform data *g* = *R*(*f*); see [4, corollary 1] for the proof.

**Theorem 2.2.**

*Let* $f\in D^{\prime }(R^{2})$ *and* *g* = *R*(*f*). *Then*

$$\left(x_{0},\xi_{0}\text{d}x)\in \mathrm{WF}(f)\;⟹\;((\theta_{0},x_{0}\cdot \omega (\theta_{0})),s\left(-x_{0}\cdot \omega {\left(\theta_{0}\right)}^{⊥}\text{d}\theta +\text{d}p\right))\in \mathrm{WF}(g\right)$$

*whenever* *θ*_0_ ∈ [0, *π*] *and s* ≠ 0 *are such that ξ*_0_ = *sω*(*θ*_0_). *Likewise, if* $(\theta_{0},p_{0})\in [0,2\pi ]\times R$ *and* q∈R, *then*

$$\left((\theta_{0},p_{0}),s(-q\text{d}\theta +\text{d}p)\right)\in \mathrm{WF}(g)\;⟹\;(x_{0},\xi_{0}\text{d}x)\in \mathrm{WF}(f)$$

*whenever x*_0_ : = *p*_0_*ω*(*θ*_0_) + *qω*(*θ*_0_)^⊥^ *and* *ξ*_0_ : = *sω*(*θ*_0_).

Note that, in theorem 2.2, *g* = *R*(*f*) is a function/distribution on some manifold M of lines in $R^{2}$. The wavefront set of *g* is therefore a subset of the tangent bundle of this manifold. Next, the theorem implies in particular that the ray transform *R* detects singularities of *f* perpendicular to a line in M. These singularities are *visible*, whereas singularities of *f* in other directions are *invisible* in the sense that they do not show up in data *g* = *R*(*f*). In particular, if *f* is a sum of characteristic functions of sets with smooth boundaries, then the tangent line to any point on such a boundary is normal to the wavefront direction of *f* at that point. Hence, a singularity of *f* at a boundary point *x* is visible if there is a line in M that is tangent to *x*.

This characterization of visible singularities can be done without reconstructing *f*. It becomes a useful tool for image reconstruction in computed tomography since this represents strong prior information about the location of edges in the image to be reconstructed. Section 5c presents an application of this principle for assessing a reconstruction. The idea is to assess performance of a reconstruction method by measuring the distance between the visible wavefront sets of the reconstruction and the theoretical one derived from the micro-canonical relation.

## 3. Shearlets and wavefront sets

Aiming for wavefront set extraction, one first realizes that the definition of the wavefront set (see definition 2.1) is very difficult to implement. In fact, it involves an unspecified localization procedure and the study of asymptotic behaviour of a localized Fourier transform. One approach to circumvent this problem is the application of representation systems from applied harmonic analysis, which allows us to define the localization procedure precisely and translate the asymptotic behaviour of the localized Fourier transform into the asymptotic behaviour of the corresponding localized coefficients.

Wavelet systems [24] can be considered the first system for singularity extraction, however, in the one-dimensional setting. Because wavelets are isotropic—i.e.not directionally aligned—they do only allow the detection of point-like structures, whereas 2D singularities are typically curvilinear structures. As discussed in §1c, shearlet systems are currently the state of the art as a system for wavefront set detection. They constitute an anisotropic representation system with the structure of an affine system, consisting of a scaling operator to change the resolution, a translation operator to change the position and a shearing operator to change the orientation, applied to a ‘mother shearlet’.

### (a) Continuous shearlets

We start by introducing continuous shearlet systems. For this, let *A*_*a*_, $a\in R^{*}:=R\setminus \{0\}$ be a *parabolic scaling matrix* *A*_*a*_, $a\in R^{*}:=R\setminus \{0\}$ and *S*_*s*_, s∈R be a *shearing matrix* given by

$$A_{a}=\left(\begin{array}{cc} a & 0\\ 0 & |a{|}^{1/2} \end{array}\right)\;\text{and}\;S_{s}=\left(\begin{array}{cc} 1 & s\\ 0 & 1 \end{array}\right).$$

Moreover, for $B\in R^{2\times 2}$, let the *dilation operator* be defined by

$$D_{B}:L^{2}(R^{2})\to L^{2}(R^{2}),\;(D_{B}f)(x)\mapsto |\det(B){|}^{-1/2}f(M^{-1}x).$$

Choosing *B* in the dilation operator as *A*_*a*_ and *S*_*s*_ yields the set of scaling and shearing operators, respectively. Let us also emphasize that the choice of shearing instead of rotation is key to allowing a faithful digitalization because the discrete versions *S*_*k*_, k∈Z, leave the digital grid $Z^{2}$ invariant. Finally, let *T*_*t*_, $t\in R^{2}$, denote the *translation operator* as defined by

$$T_{t}:L^{2}(R^{2})\to L^{2}(R^{2})\;\text{and}\;(T_{t}f)(x)\mapsto f(x-t).$$

This now leads to the following definition of continuous shearlet systems.

**Definition 3.1.**

For $\psi \in L^{2}(R^{2})$, the *continuous shearlet system* SH(ψ) is defined by

$$SH(\psi )=\{\psi_{a,s,t}:=T_{t}D_{S_{s}}D_{A_{a}}\psi =a^{-\frac{3}{4}}\psi (A_{a}^{-1}S_{s}^{-1}(\;\cdot \;\;-t)):a\in R^{*},s\in R,t\in R^{2}\}.$$

Group representation theory leads to conditions under which the associated shearlet transform is an isometry. For this, let $S:=R^{*}\times R\times R^{2}$ be endowed with the group operation

$$(a,s,t)\circ (a^{\prime },s^{\prime },t^{\prime })=(aa^{\prime },s+\sqrt{|a|}s^{\prime },t+S_{s}A_{a}t^{\prime }).$$

This is a locally compact group with a left Haar measure $d_{\mu }(a,s,t)=\text{d}a/|a{|}^{3}dsdt$.

**Theorem 3.2 ([38]).**

*Let* $\psi \in L^{2}(R^{2})$ *be admissible, i.e. it satisfies*

$$\int_{R^{2}}\frac{|\hat{\psi }(\xi ){|}^{2}}{|\xi_{1}{|}^{2}}\;\text{d}\xi <\infty .$$

*Then the continuous shearlet transform* $SH_{\psi }:L^{2}(R^{2})\to L^{2}(S)$ *given by*

$$SH_{\psi }f(a,s,t)=\langle f,\psi_{a,s,t}\psi \rangle$$

*is an isometry*.

One example for a suitable function $\psi \in L^{2}(R^{2})$ are the *classical shearlets*, which are defined by

$$\hat{\psi }(\xi )=\hat{\psi }(\xi_{1},\xi_{2})={\hat{\psi }}_{1}(\xi_{1})\;{\hat{\psi }}_{2}(\frac{\xi_{2}}{\xi_{1}}).$$

Here, $\psi_{1}\in L^{2}(R)$ is a wavelet, i.e. it satisfies the discrete Calderón condition given by

$$\sum_{j\in Z}|{\hat{\psi }}_{1}(2^{-j}\xi ){|}^{2}=1\;\text{for a.e. }\xi \in R,$$

with ${\hat{\psi }}_{1}\in C^{\infty }(R)$ and $\mathrm{supp}\;{\hat{\psi }}_{1}\subseteq [-5/4,-1/4]\cup [1/4,5/4]$, and $\psi_{2}\in L^{2}(R)$ is a ‘bump function’, namely

$$\sum_{k=-1}^{1}|{\hat{\psi }}_{2}(\xi +k){|}^{2}=1\;\text{for a.e. }\xi \in [-1,1],$$

satisfying ${\hat{\psi }}_{2}\in C^{\infty }(R)$ and $\mathrm{supp}\;{\hat{\psi }}_{2}\subseteq [-1,1]$.

**Figure 1.**
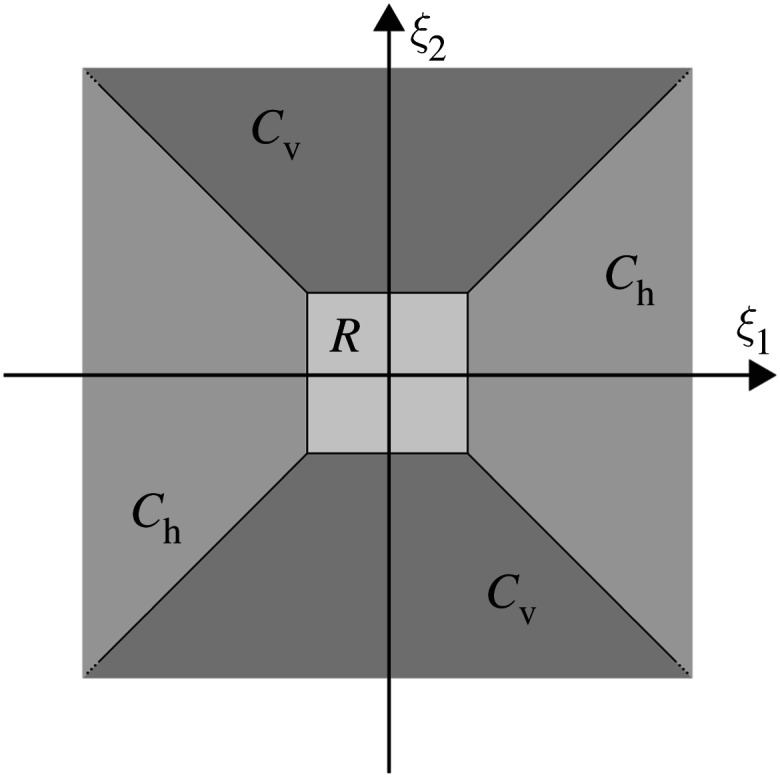
Frequency cones for the cone-adapted shearlet system.

Since the just defined continuous shearlet system exhibits a directional bias and thus in this pure form is not able to resolve any wavefront set, we require a slightly adapted version. This is based on a suitable splitting of the frequency domain into four conic regions and a low-frequency part as illustrated in figure 1. This leads to the so-called cone-adapted continuous shearlet system.

**Definition 3.3.**

For $\phi ,\psi ,\tilde{\psi }\in L^{2}(R^{2})$, the *cone-adapted continuous shearlet system* is defined by $SH(\phi ,\psi ,\tilde{\psi })=\Phi (\phi )\cup \Psi (\psi )\cup \tilde{\Psi }(\tilde{\psi })$, where

$$\begin{array}{rl} \Phi (\phi ) & =\{\phi_{t}:=\phi (\cdot -t):t\in R^{2}\},\\ \Psi (\psi ) & =\{\psi_{a,s,t}=a^{-3/4}\psi (A_{a}^{-1}S_{s}^{-1}(\;\cdot \;-t)):a\in (0,1],\;|s|\leq 1+a^{1/2},\;t\in R^{2}\},\\ \tilde{\Psi }(\tilde{\psi }) & =\{{\tilde{\psi }}_{a,s,t}:=a^{-3/4}\tilde{\psi }({\tilde{A}}_{a}^{-1}S_{s}^{-T}(\;\cdot \;-t)):a\in (0,1],\;|s|\leq 1+a^{1/2},\;t\in R^{2}\} \end{array}$$

and ${\tilde{A\;}\;}_{a}=\text{diag}(a^{1/2},a)$.

Similar to continuous shearlet systems as defined in definition 3.1, for cone-adapted continuous shearlet systems an associated transform can also be defined, namely

$$SH_{\psi }f(a,s,t):=\langle f,\psi_{a,s,t}\psi \rangle \;\text{and}\;SH_{\tilde{\psi }}f(a,s,t):=\langle f,{\tilde{\psi }}_{a,s,t}\psi \rangle ,$$

and isometry conditions can be proven [29]. With this system, we are also now able to precisely resolve a wavefront set of a distribution in the following way.

**Theorem 3.4 ([29, theorem 5.1]).**

*Let* $\psi \in L^{2}(R^{2})$ *be admissible, and* $f\in L^{2}(R^{2}).$ *Let* $D=D_{1}\cup D_{2},$ *where* $D_{1}=\{(t_{0},s_{0})\in R^{2}\times [-1,1]:$ *for* (*s*, *t*) *in a neighbourhood U of* (*s*_0_, *t*_0_), |*SH*_*ψ*_ *f*(*a*, *s*, *t*)| = *O*(*a*^*k*^) *as a* → 0, *for all* k∈N, *with the O*( · )–*term uniform over* (*s*, *t*) ∈ *U*} *and* $D_{2}=\{(t_{0},s_{0})\in R^{2}\times [1,\infty ):$ *for* (1/*s*, *t*) *in a neighbourhood U of* (*s*_0_, *t*_0_), $|SH_{\tilde{\psi }}f(a,s,t)|=O(a^{k})$ *as a* → 0, *for all* k∈N, *with the O*( · )–*term uniform over* (1/*s*, *t*) ∈ *U*}. *Then*

$$\text{WF}{\left(f\right)}^{c}=D.$$

### (b) Digital case as a semantic edge detection problem

Since we are now familiar with the approach to extract the wavefront set of a continuous distribution by analysing the asymptotic behaviour of the shearlet coefficients at a fixed position-oriented pair, we aim to extend it to real-world data. To be precise, we will now consider data coming from a finitely sampled function such as images formed by pixels representing point samples of a real-valued function. For such a signal, the notion of singularity refers to an abrupt change in intensity values, which in imaging applications typically corresponds to the presence of an edge.

In the most general case, the previously discussed approach using continuous shearlets is not directly transferable to the situation of functions being defined on a grid on a bounded domain, as the authors showed in [2]. Crudely speaking, the reason for this is the fact that we just have access to a finite number of Fourier samples as well as a finite number of shearlet coefficients. In order to overcome this limitation, we are now assuming that a digital image arises from the finite sampling of a continuous model in the sense that the image itself has a wavefront set in the sampling limit. We then aim to approximate the wavefront set by a sequence of what we call *the digital wavefront sets*.

Let us now define a digital wavefront set as introduced in [2]. In the sense of Shannon’s sampling theorem, one can make use of Paley–Wiener spaces to define a sampling space of $L^{2}(R^{2})$ for the coarsest scale Λ > 0, namely ${PW}_{\Lambda }\subset L^{2}(R^{2})$ defined by

$${PW}_{\Lambda }=:\{f\in L^{2}(R^{2}):\mathrm{supp}\left(\hat{f}\;\right)\subset {\left[-\Lambda ,\Lambda \right]}^{d}\}.$$

Using this definition of the sampled space, we now define the notion of a *digital wavefront set extractor* for the given coarsest scale Λ > 0 to be the map

3.1 $${\mathrm{DWF}}_{\Lambda }:{PW}_{\Lambda }\to P(R^{2}\times S^{1})\;\text{such that}\;\mathrm{DWF}(P_{\Lambda }f)=\mathrm{WF}(f)\text{ for all }f\in L^{2}(R^{2}).$$

The existence of a so-called faithful sequence of digital wavefront set extractors assumes that the sequence of maps ${\left\{{\mathrm{DWF}}_{j}\right\}}_{j\in N}$ converges to the continuous wavefront set extractor *WF* in the Hausdorff sense, i.e. we have

3.2 $$d_{H}(\bar{{\mathrm{DWF}}_{j}{\left(P_{j}(f)\right)}_{x}},\bar{\mathrm{WF}{\left(f\right)}_{x}})\to 0.$$

Shannon’s sampling theorem then implies that it is not possible to analytically define a sequence of digital wavefront set extractors for general function classes, which are dense on $L^{2}(R^{2})$ functions (see [2, theorem 3.3]). Although, for most classical function classes, it is impossible to construct an analytical digital wavefront set extractor on their Paley–Wiener space projections, for function classes in applications such as natural images, which are typically empirically defined, such wavefront set extractors could exist. However, these will certainly be highly sensitive to the choice of the class.

In [2], a *guiding principle* for the construction of digital wavefront set extractors served as a substitute for a rigorous mathematical definition. The most natural principle is for a wavefront set extractor to be closely adapted to the underlying function class. Typical function classes arising from real-world applications are however empirically defined. Thus most of the model-based heuristics, which could potentially be used to analytically construct a digital wavefront set extractor for each class, will fail with high probability and also limit the approach with rigid assumptions. On the other hand, although the best choice for the guiding principle will be to learn the data representation from scratch, this approach will typically be computationally intractable, requiring a lot of data and complicated architecture, such as CASENet [1] and SEAL [34].

The way out of this dilemma proposed in [2] follows the main thrust of current deep learning-based methodologies in imaging science, namely using a hybrid approach. More precisely, this approach—coined deep network shearlet edge extractor (DeNSE)—combines the model-based digital shearlet transform as a pre-processor for edge detection and a CNN that locally learns the wavefront set by classifying patches of the digital shearlet coefficients with the potential singular point at the centre. It is indeed conceivable that applying a specific, analytically defined, feature extraction such as the shearlet transform as a pre-processing step can benefit the learning process by reducing the complexity of the overall task. Intuitively, such pre-processing should perform most of the heavy lifting and should reduce the number of parameters to be learned. In fact, this approach did indeed lead to a state-of-the-art wavefront set extractor.

#### (i) Digital shearlets

Let us now delve more deeply into the digital shearlet transform [39] for a digital domain of pixel images, which—as explained before—is used in the classifier proposed in [2]. The construction of the digital shearlets is inspired by the fast wavelet transform. In fact, the digital function system can be seen as a filter bank, for which the novelty in the shearlet case resides in the definition of a faithful digital shearing operator.

To explain the digital shearlet transform in detail, let M∈N, J⊂N be finite, $k_{j}\subset N$ for all *j* ∈ *J* and $K_{j}=:[-k_{j},\ldots ,0,\ldots ,k_{j}]$. We then pick $2\sum_{j\in J}K_{J}+1$ matrices in $R^{M\times M}$, and denote these matrices by *ϕ*^dig^ and $\psi_{j,k,\iota }^{\text{dig}}$ for $j\in J,k\in K_{j},\iota \in \{-1,1\}$. To make the connection to the continuous shearlet transform, we can think of $\psi_{j,k,\iota }^{\text{dig}}$ as a digitized version of $\psi_{2^{-j},2^{-j/2}k,0,\iota }$ and of *ϕ*^dig^ as a digitized version of a low-frequency filter. An explicit—and highly technical—construction of the matrices *ϕ*^dig^ and $\psi_{j,k,\iota }^{\text{dig}}$ can be found in [39]. These are then exploited to define the *digital shearlet transform* of an image $I\in R^{M\times M}$ by

$$\mathrm{DSH}(I)(j,k,m,\iota )=:\left\{ \begin{array}{ll} \langle I,T_{m}\psi_{j,k,\iota }^{\text{dig}}\rangle , & \text{if }\iota \in \{-1,1\},\\ \langle I,T_{m}\phi^{\text{dig}}\rangle , & \text{if }\iota =0, \end{array}\right.$$

where *j* ∈ *J*, *k* ∈ *K*_*j*_, $m\in {\left\{1,\ldots ,M\right\}}^{2}$, and $T_{m}:R^{M\times M}\to R^{M\times M}$ circularly shifts the entries of the elements of a matrix by *m*. Thus, from a structural viewpoint, the digital shearlet transform of an image $I\in R^{M\times M}$ is a stack of $2\sum_{j\in J}(K_{j}-1)+1$ matrices of dimension *M* × *M*. We will refer to this stack of images as the shearlet volume.

#### (ii) DeNSE algorithm

Using the digital shearlet transform as the input of a CNN classifier, the DeNSE algorithm is able to perform wavefront set extraction with high accuracy and closely adapted to the class of the training data. In addition to following the guiding principle, the main purpose of this approach was to achieve high accuracy concerning the task of wavefront set extraction for inverse problem regularization. With this in mind, the problem of classifying pixels of a digital image into *N* + 1-classes, where *N* of these classes correspond to orientations of singular points and the additional (*N* + 1-th) class is the binary class of a pixel being an edge, is subdivided into *N* + 1 separate binary classifiers. Note that pixels corresponding to edges will have at least two class labels, namely one orientation class and one edge/non-edge class. Some pixels such as the corner points will have more than two class labels. Each pixel will be classified independently by using a patch-based approach in the sense that the patch of the shearlet coefficients, associated with the image-spaced patch centred at this pixel, is analysed with respect to the occurrence of an edge in this pixel. Let us now be more specific, as follows.

**Figure 2.**
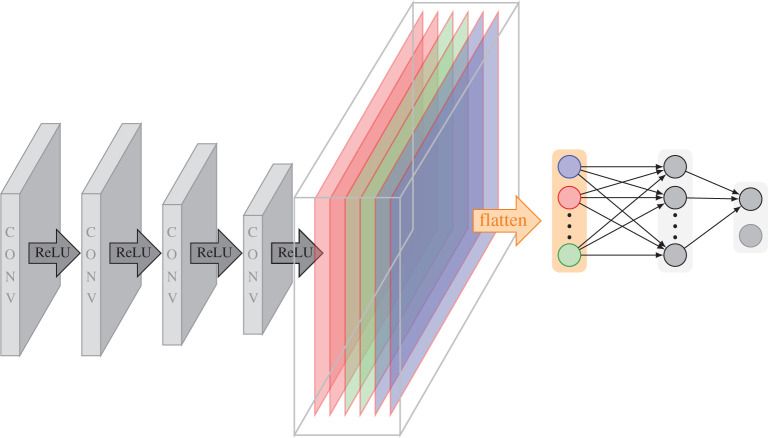
Illustration of the DeNSE network architecture, consisting of four convolutional layers and one fully connected layer. The coloured block in the middle represents a stack of the output of the last convolutional layer. The colours correspond to the different channels. (Online version in colour.)

The architecture used for each classification is composed of four convolutional layers, with 2 × 2 max pooling, ReLU activation (ReLU(*x*) = max{*x*, 0}) and batch normalization, followed by a fully connected layer with 1024 neurons, softmax activation function and a one-dimensional output. This architecture was shown to perform well in a series of tests while being of moderate size. The network architecture is illustrated in figure 2.

The input of the DeNSE architecture are small patches of shearlet coefficients in the following sense. Let, for instance, *J* = 4 be the coarsest scale, with the corresponding shearing levels for each scale given by $K_{j}=2^{\left\lceil j/2+1\right\rceil }+1$. Then the digital shearlet coefficient *DSH*(*I*) will form a three-dimensional array composed of *L* stacked images of the same size as the original image, where $L=2\sum_{j\in J}(K_{j}-1)+1=49$. These can be computed via Julia API of the software ShearLab [27] (www.shearlab.org/software). Considering, for example, the choice for the size of the patches to be 21 × 21 as in [2], the input of DeNSE will then be a stacked set of patches of size 21 × 21 × 49. Assuming that we consider 180 orientations ${\left\{\theta_{i}\right\}}_{i=1}^{180}$, the output will be one element of the 180 classes, where the centre point of the patch belongs.

Let us now recall the training procedure. For each *θ*_*i*_, *i* = 1, …, 180, the network is trained on patches of shearlet coefficients of images $I\in R^{M\times M}$ of the form

3.3 $${\left(\mathrm{DSH}(I)(j,k,m,\iota )\right)}_{j\in J,k\in K_{j},\iota \in \{-1,0,1\},m\in [m_{1}^{*}-10,m_{1}^{*}+10]\times [m_{2}^{*}-10,m_{2}^{*}+10]},$$

with m* ∈ {11, …, *M* − 10}^2^ being the centre point of the patch to which the classified orientation is assigned. By using a softmax binary classifier, the label associated with a batch of (3.3) is 1 if *I* has a singularity with orientation *θ*_*i*_ at m*. In addition, there exists a separate classifier, which uses the same data and assigns 1 to a patch whose central pixel corresponds to a singular point (edge point). Although each image in the training set is normalized before computing the shearlet coefficients, no further pre-processing of the input patches was performed. The code to reproduce the experiments can be downloaded from https://www3.math.tu-berlin.de/numerik/www.shearlab.org/applications.

The DeNSE algorithm was in fact shown to outperform other methods, for example CASENet and SEAL, on the standard datasets such as BSDS500 (Berkeley segmentation dataset) with 503 natural images, the SBD with 11 355 natural images and a set of phantoms formed by ellipses and parallelograms with analytically defined wavefront sets. We present some of these results in §5.

### (c) Avoiding the distracted supervision paradox

The distracted supervision paradox as introduced in §1d refers to the fact that semantic edge detection such as wavefront set extraction requires the supervision of two fundamentally different tasks:

— *Category-agnostic edge detection* requires the detection of pixels corresponding to edges and other singularities, and thus the use of low-level features.
— *Semantic edge classification* requires the classification of edges with abstracted high-level semantics, and therefore relies on high-level features.

Intuitively accomplishing both tasks jointly seems infeasible as both problems require very different features. Yu *et al.* [1] confirmed with the CASENet architecture that a naive joint supervision of both tasks performs less well than directly learning the semantic edges with no deep supervision that combines both features.

This paradox imposes an upper bound on the performance of methods that are aiming to directly learn (in an end-to-end fashion) semantic edges. Since the initial work [1], there have been several approaches to avoid this paradox. One particularly remarkable approach is the one presented by Liu *et al.* [33]. In their paper, they propose a network architecture containing a backbone based on residual CNNs—similar to the CASENet, but with the introduction of novel information converter layers; this allows information coming from lower levels to be combined for edge supervision with information from higher levels used for semantic supervision. This approach successfully established a new state of the art in semantic edge detection, with a significant performance improvement.

The key to the DeNSE algorithm resides in the splitting of the multi-label classification task into individual binary classifiers inspired by the performance increment. In addition, DeNSE separates the category-agnostic edge detection and the semantic edge classification, which already avoids the distracted supervision paradox.

It is important to mention that CASENet was designed to perform general semantic edge detection, whereas DeNSE is a specialized model for wavefront set extraction, which is also a semantic edge detection task but purely local. In that sense, DeNSE outperforms CASENet in wavefront set extraction, but DeNSE cannot be used for non-local semantic edge detection. In the next section, we present our attempt to extend the main ideas behind the design of DeNSE for non-local semantic edge detection.

## 4. General semantic edge detection using shearlets and deep supervision

The use of shearlets for achieving high precision in digital wavefront set extraction, with significantly better results than the general approach CASENet (table 1), motivates the introduction of the shearlet transform in general semantic edge detection, where classes of edges typically come from the particular object the edge belongs to. In order to exploit the power of the shearlet transform as a pre-processing step, we will use the backbone of the current state-of-the-art approach on general semantic edge detection namely the CASENet architecture [1]. As already mentioned, after the introduction of CASENet and the distracted supervision paradox, most of the approaches to performing semantic edge detection used the same architecture backbone because of its effectiveness. Aiming to take the best of both worlds, we will now introduce a hybrid method which can tackle any semantic edge detection problem while retaining the efficiency of the shearlet-based DeNSE approach.

**Table 1.** Performance of wavefront set extraction on the head-phantom dataset. All values are percentages. Italic indicates work by the current authors.

method	MF-score
Yi–Labate–Easley–Krim [40]	75.7
CoShREM [23]	70.4
CASENet [1]	78.6
SEAL [34]	83.4
DDS [33]	85.6
*DeNSE* [2]	*95.7*

In the following, we explore two alternative architectures, namely the original CASENet and the deep diverse supervision approach presented by Liu *et al.* [33], where information converters are used for the joint training of low-level edge supervision and high-level edge classification. In order to show the heavy lifting that the shearlet transform is able to do, we use as the input for these networks the shearlet coefficients of the images, and extend the channels of the first convolutional layer by the number of slices of the particular shearlet coefficients. We also decrease the depth of the resulting network by removing the last category-agnostic edge feature map ResNet subnetworks, resulting in an overall smaller network, which will be detailed in a follow-up paper.

### (a) Shear-CASENet: deep shearlet category-aware semantic edge detection

**Figure 3.**
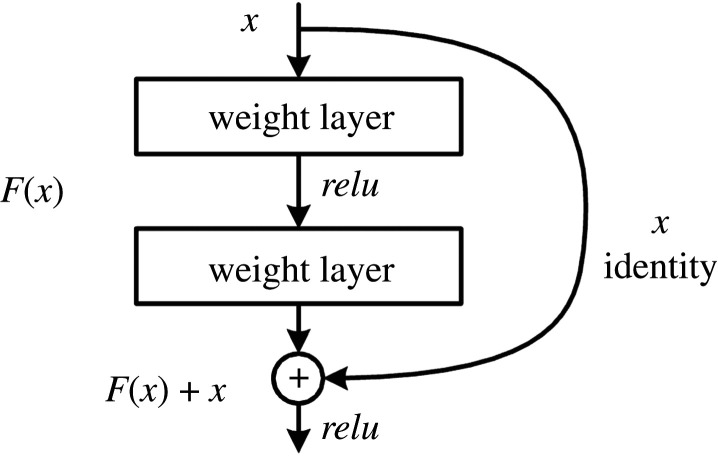
Illustration of the principal block in ResNet, namely the skip connection from the input to the output is the main characteristic of this architecture (taken from https://neurohive.io/en/), where *relu*(*x*) = max{*x*, 0}.

The CASENet architecture [1] is based on the already known residual neural network architecture, known as ResNet (figure 3). This architecture has shown tremendous success in different image-processing tasks, including image classification. In fact, it won the ImageNet challenge in 2015. CASENet receives as input the image and outputs a 2D array of the same size with the classified edges represented as pixels with value given by the corresponding category.

**Figure 4.**
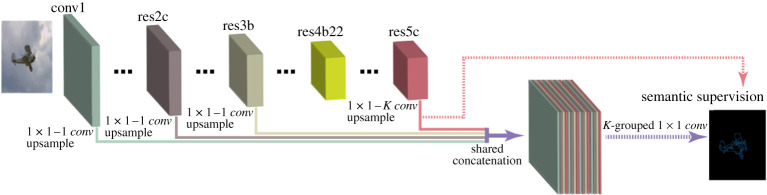
Illustration of the classical CASENet architecture (inspired by the figures in [33]). (Online version in colour.)

We next explain the CASENet architecture, which is displayed in figure 4, in more detail. The input image is connected to a 1-channel convolutional layer (conv1), which is followed by four stacked ResNet subnetworks: res2c, res3b, res4b22 and res5c. Each of these subnetworks is a block of the network ResNet-101, where res(N)(M) represents the M-th layer (represented by the letter ‘a’, ‘b’ and ‘c’) of the N-th stage of ResNet-101. In addition, stage 4 of ResNet-101 has more than three layers, so it has a different naming system from the other stages, where its first layer is named as ‘a’ and the rest are all ‘b’s’. The layer res4b22 is the 23rd layer of that stage.

The first three stages of CASENet (i.e. conv1, res2c, res3b) produce a single-channel feature map *F*^(*m*)^, which is used to perform the edge detection part. The last stage, res5c, is connected to a 1 × 1 convolutional layer to produce a *K*-channel class activation map $A^{\left(5\right)}=\{A_{1}^{\left(5\right)},A_{2}^{\left(5\right)},\ldots ,A_{K}^{\left(5\right)}\}$, where *K* is the total number of categories. In order to combine the edge information coming from the first stages, at the end of the network one replicates the bottom features *F*^(*m*)^ by concatenating them in each channel of the class activation map at the last stage; more precisely,

$$A^{f}=\{F^{\left(1\right)},F^{\left(2\right)},F^{\left(3\right)},A_{1}^{\left(5\right)},\ldots ,F^{\left(1\right)},F^{\left(2\right)},F^{\left(3\right)},A_{K}^{\left(5\right)}\}.$$

At the end, a *K*-grouped 1 × 1 convolutional layer is applied to *A*^*f*^, generating a semantic edge map with *K* channels, where the *k*-th channel represents the edge map for the *k*-th category. Summarizing, the first four stages of CASENet produce category-agnostic edge feature maps with different levels of refinement. This depth becomes necessary to produce edges fine enough to be classified by the last stage.

**Figure 5.**
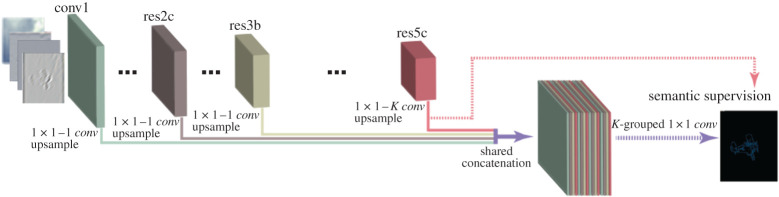
Illustration of the shear-CASENet architecture (inspired by the figures in [33]). (Online version in colour.)

Following our goal to develop a hybrid approach, we introduce the deep shearlet category-aware semantic edge detection (shear-CASENet) architecture, which takes as input the shearlet coefficients of an image (interpreted as channels) and produces as output an array with the same size as the image with the classified edges. It uses the same backbone as the original architecture but removes the fourth stage. In figure 5, we depict the proposed architecture. This architecture will take advantage of the representation power given by the shearlet transform for edge detection to increase the performance and efficiency of the CASENet architecture.

### (b) Shear-DDS: shearlet diverse deep supervision

Although the CASENet architecture led a new state of the art in semantic edge detection, it has its limitations. One fundamental limitation is the joint training, with deep supervision, of the category-agnostic edges and the edge classification, which the authors coined distracted supervision paradox. In CASENet, even the fourth stage is not used in the supervision. It was in fact introduced as a way to alleviate the information conflicts coming from the first three stages and the fifth stage, also known as a buffer unit.

In more recent years, different alternatives to train the network have been introduced. Yu *et al.* [34] developed the simultaneous edge alignment and learning (SEAL) architecture, which simultaneously aligns the ground truth edges and learns the corresponding classifier, with the downside being that it is time consuming because of the necessary CPU usage by the alignment step. Recently, Liu *et al.* [33] introduced a novel way to train CASENet, also known as the deep diverse supervision. This approach makes use of an information converter based on a convolutional residual block (figure 3), where the output of each stage is fused in a final shared concatenation. The information converters help to assist low-level feature learning (stages 1–4) in order to generate consistent gradient signals from the higher levels (stage 5), producing a highly discriminative feature map for high-performance semantic edge detection. It is worth noting that, in this case, stage 4 is no longer a buffer, but is used in the supervision. Having the category-agnostic edge maps obtained from the information converter applied to each of the first four stages, namely *E* = {*E*^(1)^, *E*^(2)^, *E*^(3)^, *E*^(4)^}, the final map will be given by the information conversion of the fifth stage and the shared concatenation, i.e.

$$E^{f}=\{E,A_{1}^{\left(5\right)},E,A_{2}^{\left(5\right)},\ldots E,A_{K}^{\left(5\right)}\}.$$

**Figure 6.**
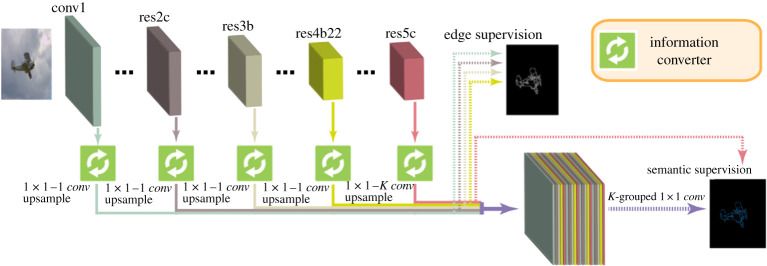
Illustration of the classical diverse deep supervision architecture (inspired by the figures in [33]). (Online version in colour.)

Figure 6 depicts the resulting architecture.

This network is trained with a multi-task loss in the sense that two different losses, corresponding to category-agnostic and category-aware edge detection, are optimized jointly. Both losses are based on a re-weighted sigmoid cross-entropy loss, which is typically used for multi-label classification. For further details, we refer to [33].

**Figure 7.**
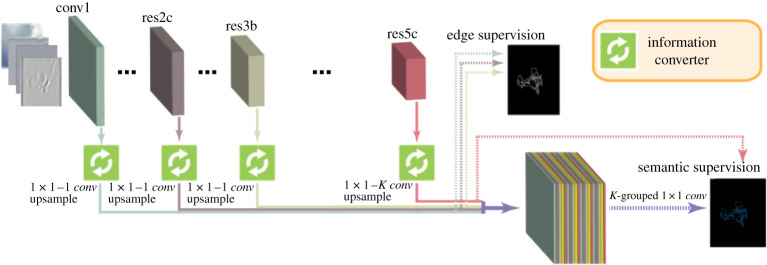
Illustration of the shearlet diverse deep supervision architecture (inspired by the figures in [33]). (Online version in colour.)

Inspired by the construction of shear-CASENet, we introduce the shearlet-based diverse deep supervision (shear-DDS) architecture. This architecture will accept as input the shearlet volume of an image, with the same backbone as DDS but without the fourth stage. The output consists of the same activation map characterizing the classified edges as the original DDS. Figure 7 shows the new shear-DDS architecture.

Shear-DDS was trained with the same multi-task loss function as the original architecture, obtaining an improvement in performance on the SBD dataset. The corresponding numerical results are presented in §5b.

### (c) Complexity and performance of shear-CASENet and shear-DDS

We now turn to an analysis of the complexity and performance of shear-CASENet and shear-DDS, particularly in comparison with CASENet and DDS, respectively, thereby showing the power of the pre-processing with shearlets.

We start with shear-CASENet, first analysing the number of trainable parameters and showing that shear-CASENet has significantly fewer classical CASENet. Indeed, if the input is an *N* × *N*-image, the fourth stage of CASENet will have 1024 × *N*^2^/4 parameters. By having that stage removed and extending the first convolutional layer by *L* shearlet channels, one obtains a reduction of (128 − *L*) × *N*^2^ trainable parameters. Typically four scales in the shearlet transform are used, i.e. *L* = 49, leading to an image of size 256 × 256. Shear-CASENet thus has 37 259 387 parameters, while the classical CASENet has 42 436 731, yielding around 13% fewer parameters to train.

Second, we turn to the storage complexity. For this, note that each coefficient image is given by the convolution of a shearlet filter and the input image. Using the associative property of the convolution, we can write the first layer of the shear-CASENet as *L* separate convolutions with the partially trainable kernels ${\left\{{\tilde{\omega }}_{l}\right\}}_{l=1}^{L}$ given by ${\tilde{\omega }}_{l}=\psi_{l}*\omega_{l}$ for *l* ≤ *L*, where *ψ*_*l*_ is the *l*-th (non-trainable) shearlet filter and *ω*_*l*_ is a trainable convolutional kernel. The reduction in the number of trainable parameters in the architecture by (128 − *L*) × *N*^2^ implies a reduction of 128 − *L* trainable convolutional kernels to store and the same number of convolutions to compute. At the same time, we still need to store *L* non-trainable shearlet filters, and compute an additional *L* convolutions. Since four scales is typically sufficient, this results in *L* = 49 shearlet filters. Because 128 − *L* = 79 > 49 = *L*, the shear-CASENet architecture involves 128 − 2*L* = 30 fewer convolutional kernels to store.

In addition, the reduction in the overall convolutional kernels and trainable parameters also reduces the computational complexity of the end-to-end model, since the total number of basic computations (convolutions) is reduced.

Summarizing, shear-CASENet leads to a significant decrease in computational complexity and memory requirements with respect to its non-shearlet counterpart CASENet, even taking the pre-processing into account. As will be shown in §5b for the SBD (http://home.bharathh.info/pubs/codes/SBD/download.html) as the benchmark database, shear-CASENet also performs significantly better for semantic edge detection than CASENet.

Finally, similar arguments applied to the case of shear-DDS show that we achieve a 13% reduction in trainable parameters, in memory usage, and in computational complexity compared with DDS for this approach as well. As before, shear-DDS also shows significantly improved performance with respect to its non-shearlet version; table 2 depicts these results.

**Table 2.** Semantic edge detection performance on the SBD dataset. All values are percentages. Italic indicates work by the current authors.

method	MF-score
DSN [1]	65.2
SEAL [34]	75.3
classical CASENet [1]	71.4
classical DDS [33]	78.6
*shear-CASENet*	75.7
*shear-CASENet 2*	75.9
*shear-DDS*	80.1
*shear-DDS 2*	80.4

## 5. Numerical results and applications

To show the true impact of the approach presented in this work, we performed several numerical experiments, targeted at three specific applications; namely, wavefront set extraction, general semantic edge detection and computed tomography reconstruction. In each of the experiments, the networks were trained and evaluated on datasets for the specific purpose. On one hand, the general semantic edge detection application was trained and evaluated on the SBD, which is a standard benchmark for this application. On the other hand, since wavefront set extraction is well suited to being used in computed tomography reconstruction (see §2c), the DeNSE subnetworks were trained on images formed by random ellipses which resemble human-head phantoms such as the well-known Shepp–Logan phantom.

All experiments show that our hybrid approach, namely combining shearlets with carefully designed network architectures, provides a significant improvement in performance. We thus expect that this conceptual approach should be beneficial for other image-processing tasks as well.

### (a) Wavefront set extraction

For wavefront set extraction, we used the TensorFlow implementation of the DeNSE architecture, publicly available from http://www.shearlab.org/applications. We used the TensorFlow interface of the Julia API of ShearLab, available from http://www.shearlab.org/software, to compute the shearlet transform of TensorFlow tensors; we refer to [41] for the details on such tensor implementation.

As the training dataset, we used head-like phantom images, inspired by the Shepp–Logan phantom, consisting of a specific selection of random ellipses, two large ellipses, representing the inner and outer skull, and small ellipses representing inside the skull, with different sizes, pixel intensity value and orientations. We also varied the gradient of the intensity of the ellipses in order to obtain curves with different levels of regularity. The advantage of using this type of phantom is twofold: it allows access to the analytical wavefront set and the resulting network can be used for computed tomography applications in head-like phantoms.

We then trained the DeNSE architecture with the procedure presented in [2]. The DeNSE architecture classifies patches of an image for a specific given orientation. We used a resolution of 180 distinct orientations and trained the network for each orientation separately. For each training set, we used 10 000 images. The evaluation and test were then done over 2000 images each. We made use of a total of four scales on the shearlet transform, producing a shearlet coefficient volume with 49 slices. From the shearlet coefficient of each image, we extracted 10 distinct patches randomly. This was done in such a way that the classes were balanced, meaning that, in each binary classifier, the number of positive cases is roughly the same as the number of negative cases.

As a performance measure for the classification, we used the standard MF-score, which is the mean of the F-score over all the orientations. We compared the performance with other semantic edge detection models, making use of the publicly available Python code for CoShREM [23], CASENet [1] and SEAL [34]. For the Yi–Labate–Easley–Krim [40] and DDS [33] models, we used our own implementation. The performance benchmarks with these models are presented in table 1.

**Figure 8.**
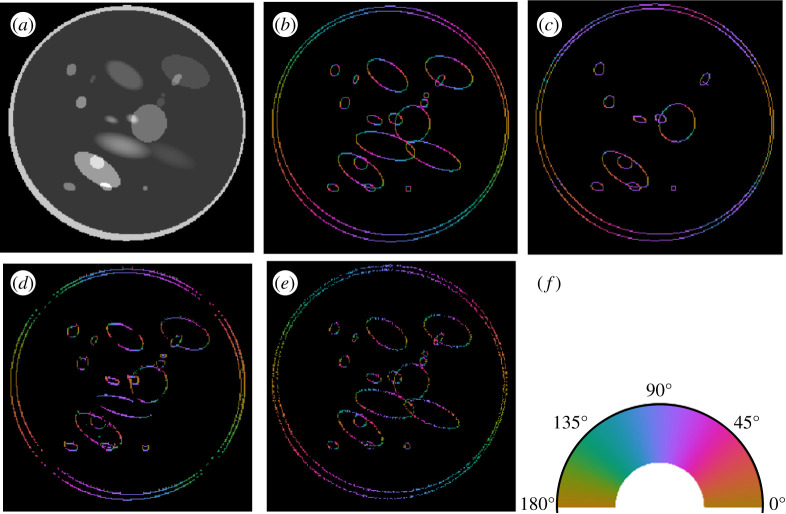
Computed edges and orientations of an example of the head-phantom dataset. (*a*) Input image. (*b*) Orientations, analytical ground truth. (*c*) Orientations predicted by the CoShREM algorithm. (*d*) Orientations predicted by the CaSENet. (*e*) Orientations predicted by the DeNSE algorithm. (*f*) Colour code for normal-directions. (Online version in colour.)

Figure 8 provides a visual comparison of the results of the wavefront set extraction on an example of the head-phantom dataset using three different models, the CoShREM model and the CASENet architecture. Judging from the images, it is clear that DeNSE shows significantly better performance than CoSHREM—the latter is not even able to find the ellipses with smooth boundaries.

### (b) General semantic edge detection

For the numerical experiments of the general case of semantic edge detection, we trained the shear-CASENet and shear-DDS architectures on the SBD (http://home.bharathh.info/pubs/codes/SBD/download.html). This database consists of 11 135 images, from which we used 9035 images for training, 1050 for evaluation and 1050 for testing. Each image has a human-annotated array of edge-pixels with the intensity value as the category number of the object that this edge belongs to. The SBD dataset consists of a total of 20 categories, including vehicles, animals and plants.

Both shear-CASENet and shear-DDS were trained on the full shearlet coefficients computed on normalized images, without further preparation. Similar to the case for the wavefront set extraction, we used the digital shearlet transform [39] implemented in TensorFlow (https://github.com/arsenal9971/tfshearlab), with a total of four scales. This produces a shearlet coefficients volume of 49 slices, which was then fed into the proposed architectures. We remark that performing the convolution with the shearlet filters implemented in TensorFlow allowed us to use the same GPU-acceleration capacities of TensorFlow to obtain a reduction in the running time with respect to their non-shearlet counterparts.

As implementation, for the CASENet architecture we used the publicly available version from https://github.com/lijiaman/CASENet. This implementation uses the deep learning framework PyTorch, thereby making it compatible with our shearlet implementation. Based on this code, we implemented the deep diverse supervision architecture by introducing the information converters and the proposed multi-task loss. Also based on this code, we implemented the shear-CASENet and shear-DDS architectures by extending the first convolutional layer with the corresponding shearlet channels (figures 5 and 7) and removing the fourth stage of the original architectures. Our code is publicly available at https://www3.math.tu-berlin.de/numerik/www.shearlab.org/applications.

For comparison purposes, in addition to comparing our approach with CASENet and DDS, we also included a comparison with the deeply supervised version of CASENet [1] and SEAL [34]. The performance benchmarks presented in table 2 are in terms of the MF-score, in a similar fashion to the wavefront set extraction benchmarks, by computing the MF-score over all the categories. It is clearly visible that the MF value is slightly better for shear-CASENet and shear-DDS than the other architectures. The improvement is not as significant as we observed for the task of wavefront set extraction, the reason being that DeNSE was specifically designed for this task and the existing models have general semantic edge detection applications. It is worth stressing, though, that shear-CASENet and shear-DDS have significantly fewer parameters than their non-shearlet counterparts.

In addition, we have also trained the shearlet-based networks without removing the fourth stage (buffer layer) to study how relevant such a layer is to the modified architecture. Table 2 shows that adding the fourth layer will not improve the performance significantly. It will in fact contain even more parameters than the original non-shearlet architectures. In the original work, the fourth stage was mainly used as a buffer layer to alleviate the information conflict coming from the distracted supervision paradox. Our results suggest that, by introducing the shearlet representation, we alleviate these conflicts, mainly because of the improvement in edge localization given by the shearlet representation.

**Figure 9.**
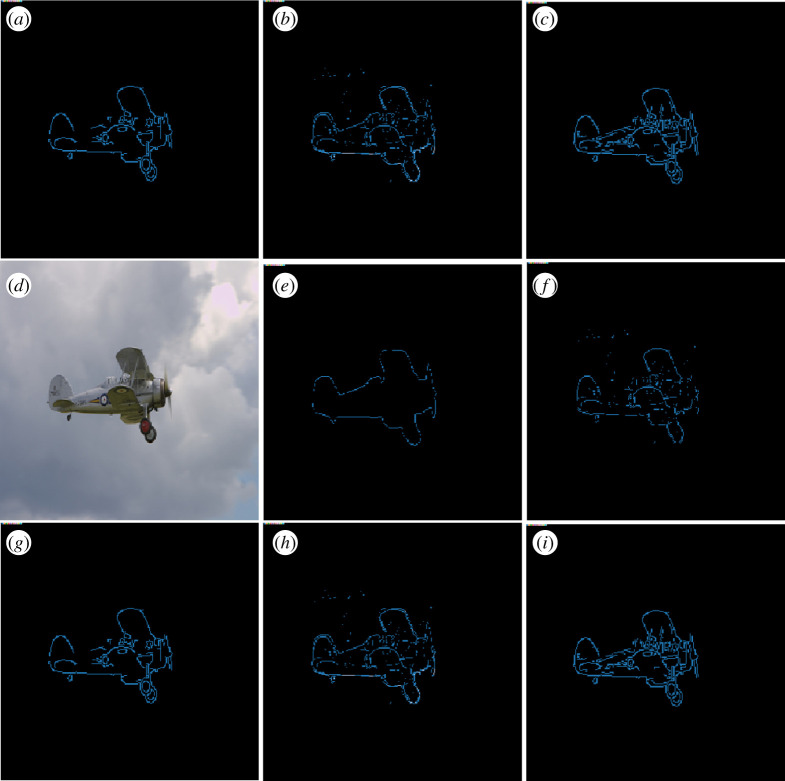
Computed semantic edges of an example of the SBD. The colour blue represents the category of aeroplane. (*a*) Input image. (*b*) Semantic edges, human annotation. (*c*) Semantic edges predicted by the classical CASENet architectures. (*g*) Semantic edges predicted by the classical DDS architecture. (*h*) Semantic edges predicted by the shear-CASENet architecture. (*i*) Semantic edges predicted by the shear-DDS architecture. (Online version in colour.)

**Figure 10.**
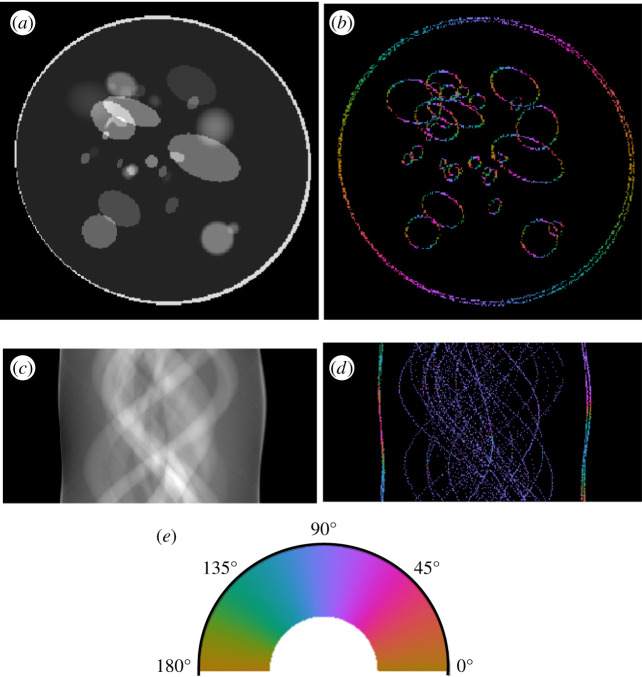
(*a*) Phantom made from ellipses. (*b*) Associated wavefront set obtained by the inverse canonical relation on the wavefront set of the low-dose sinogram. (*c*) Low sinogram, with every six angles measured. (*d*) Associated wavefront set obtained by DeNSE. (*e*) Colour code for normal directions. (Online version in colour.)

For visual comparison, in figure 9, we depict the results obtained using an example of the SBD dataset. It shows the semantic edges obtained by both CASENet and DDS and their respective shearlet extension. In all cases, the aeroplane in the picture was correctly classified, but the refinement of the obtained edges is clearly improved in the shearlet version. This strongly suggests that the use of shearlets is well suited for high performance in semantic edge detection (Figure 10).

### (c) Tomographic reconstruction

As shown in §2c, microlocal analysis can be used to describe how the planar ray transform modifies wavefront sets (singularities). To show this in the context of digitized images and data, we make use of the digital wavefront set extraction applied to the head-phantom dataset introduced in §5a and the microlocal canonical relation of the ray transform (§2c), in order to obtain the digital wavefront set an image from the digital wavefront set of its sinogram without a previous inversion. This approach is based on the work presented by the authors in [2]. We simulate tomographic data (sinograms) using the Python implementation of the digital ray transform in the operator discretization library (http://github.com/odlgroup/odl).

Let us now explain the training procedure. To label each sinogram with the correct wavefront set, we used a digitized version of the canonical relation for the ray transform, presented in [2, definition 6.1]. This definition was then taken as an ansatz for the definition of the digital wavefront set of the sinograms, which came from a phantom whose wavefront set we know explicitly. We then trained the DeNSE model on the sinogram wavefront sets, with the same training, test and evaluation set as in §5a. Using the results of the canonical relation as the ground truth for the sinogram wavefront sets, we obtained an MF-score over the test dataset of 95.7%, which is comparable to the performance of DeNSE on the head-phantom image class. We will not present any performance comparisons, since we are not aware of any competing algorithm for the detection of a wavefront set on sinograms.

Since applying the inverse canonical relation to the wavefront set of the sinogram will give us access to part of the wavefront set of the original image, without performing any inversion, having a method to detect the wavefront set of a sinogram becomes useful for inverse problem regularization.

**Figure 11.**
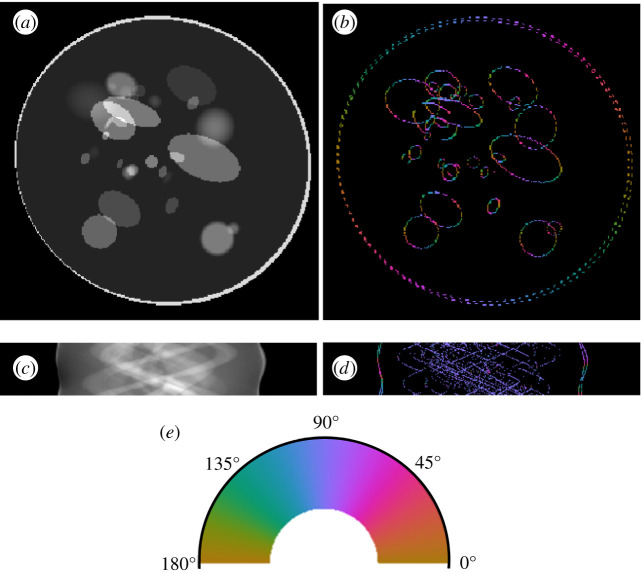
(*a*) Phantom made from ellipses. (*b*) Associated wavefront set obtained by the inverse canonical relation on the wavefront set of the low-dose sinogram. (*c*) Low sinogram, with every six angles measured. (*d*) Associated wavefront set obtained by DeNSE. (*e*) Color code for normal directions. (Online version in colour.)

To show this potential, we present in figure 11 the phantom wavefront set obtained when applying the inverse canonical relation to the low-dose sinogram and measuring every 6^°^. This low-dose problem is highly ill-posed, and will require significant effort to be inverted. Not having at hand the digital canonical relation will give us almost for free the part of the phantom’s wavefront set associated with the measured angles.

To further show the use of this method, we performed a low-dose sinogram example, three standard inversion schemes, filtered back-projection, Tikhonov regularization and total variation regularization. We first compute the associated image reconstruction from the low-dose sinogram, measured every six angles, and then compute the associated wavefront set of the reconstruction with DeNSE. We next compute the average mean square error of the true wavefront set of the data point. Via the inverse canonical relation, we compare this with the error resulting from computing the wavefront set of the sinogram and mapping it back to the image.

**Figure 12.**
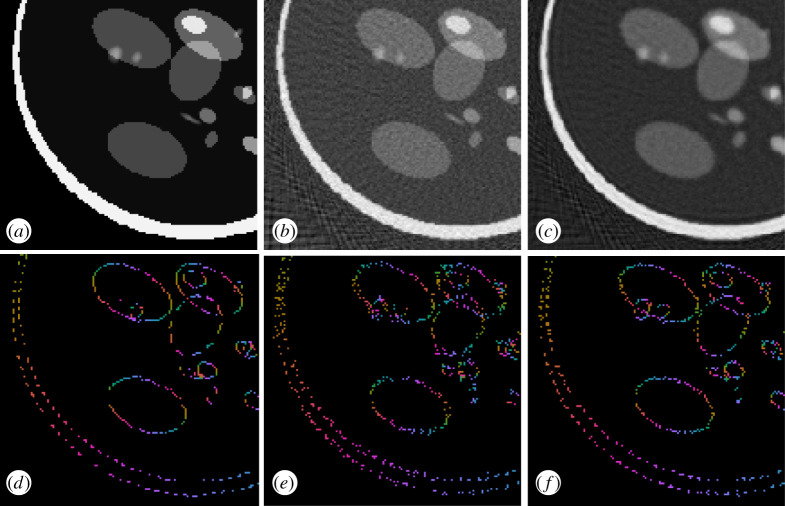
(*a*) Phantom made from ellipses. (*b*) Filtered back-projection reconstruction. (*c*) Tikhonov reconstruction. (*d*) Wavefront set of the phantom data computed by the inverse canonical relation on the low-dose sinogram wavefront set extracted by DeNSE. (*e*) Wavefront set of the filtered back-projection reconstruction extracted by DeNSE. (*f*) Wavefront set of the Tikhonov reconstruction extracted by DeNSE. (Online version in colour.)

Figure 12 shows the wavefront sets corresponding to the different reconstruction schemes. In table 3, the obtained errors are presented, clearly showing the advantage of first extracting the wavefront set of the sinogram and then applying the canonical relations, over any first-invert-then-extract strategy. In addition, this *a priori* information can be used as a regularizer for any variational regularization scheme.

**Table 3.** Error of wavefront set estimation by different inversion techniques.

inversion technique	mean square error
Tikhonov	443.0
total variation	380.9
filtered back-projection	504.3
canonical relations	168.1

## 6. Discussion of numerical experiments and conclusion

In the numerical experiments, we observed an improvement in the performance of edge extraction and classification when using the shearlet transform for model-based feature extraction before feeding the network. This approach is inspired in the DeNSE model [2] for wavefront set extraction, which has shown a significant improvement with respect to the traditional methods designed for wavefront set extraction [23,40], owing to the combination of both the model-based shearlet representation and the data-driven high-performance classification, as well as modern deep learning-based models [1,33,34]. We have shown in this work that such a method is a particular case of semantic edge detection and, by design, is able to successfully avoid the distracted supervision paradox.

For general semantic edge detection, introducing a change of representation systems to the shearlet system improved the performance of the standard semantic edge detection models (CASENet and DDS). The results also suggest that the use of shearlets in image-processing tasks involving edge detection helps to do heavy-lifting in a model-based fashion, reducing the amount of parameters needed and resulting in a reduction in complexity. This is clearly shown by the shear-CASENet and shear-DDS architecture, which perform better than their non-shearlet version, but have fewer learnable parameters as well as reduced computational and storage complexity. This combination of model-based and data-driven approaches is a strategy that more researchers have adopted in the last few years.

Finally, we have also observed that the removal of the fourth block in the proposed architectures does not have a significant impact on its performance, suggesting that the shearlet representation alleviates the training conflicts caused by the distracted supervision. With this in mind, we can conclude that the shearlet representation improves not only the edge detection of the semantic edge task but also the semantic classification.

In addition, the applications of semantic edge detection in inverse problems have not yet been explored in depth but the results presented in §5c are a clear indication that this area will be fruitful in the next few years. As part of the future developments, the authors will work on the use of the wavefront set extractor to improve the existing hybrid methods on tomographic reconstruction.

## Data accessibility

The code to reproduce the experiments can be downloaded from https://www.shearlab.org/applications.
